# Guiding functional connectivity estimation by structural connectivity in MEG: an application to discrimination of conditions of mild cognitive impairment

**DOI:** 10.1016/j.neuroimage.2014.08.002

**Published:** 2014-11-01

**Authors:** José Angel Pineda-Pardo, Ricardo Bruña, Mark Woolrich, Alberto Marcos, Anna C. Nobre, Fernando Maestú, Diego Vidaurre

**Affiliations:** a Laboratory of Neuroimaging, Centre for Biomedical Technology, Universidad Politécnica de Madrid, Campus de Montegancedo, 28223 Pozuelo de Alarcón, Spain; b Laboratory of Cognitive and Computational Neuroscience, Centre for Biomedical Technology, Universidad Politécnica de Madrid, Campus de Montegancedo, 28223 Pozuelo de Alarcón, Spain; c Oxford Center for Human Brain Activity (OHBA), University of Oxford, Oxford, United Kingdom; d The Oxford Centre for Functional MRI of the Brain (FMRIB), University of Oxford, Oxford, United Kingdom; e Department of Neurology, Hospital Clínico San Carlos, Madrid, Spain

**Keywords:** Resting state, Diffusion tensor imaging, Magnetoencephalography, Multimodal neuroimaging, Multivariate sparse regression, Graphical Lasso, Mild cognitive impairment, Machine learning

## Abstract

Whole brain resting state connectivity is a promising biomarker that might help to obtain an early diagnosis in many neurological diseases, such as dementia. Inferring resting-state connectivity is often based on correlations, which are sensitive to indirect connections, leading to an inaccurate representation of the real backbone of the network. The precision matrix is a better representation for whole brain connectivity, as it considers only direct connections. The network structure can be estimated using the graphical lasso (GL), which achieves sparsity through *l_1_*-regularization on the precision matrix. In this paper, we propose a structural connectivity adaptive version of the GL, where weaker anatomical connections are represented as stronger penalties on the corresponding functional connections. We applied beamformer source reconstruction to the resting state MEG recordings of 81 subjects, where 29 were healthy controls, 22 were single-domain amnestic Mild Cognitive Impaired (MCI), and 30 were multiple-domain amnestic MCI. An atlas-based anatomical parcellation of 66 regions was obtained for each subject, and time series were assigned to each of the regions. The fiber densities between the regions, obtained with deterministic tractography from diffusion-weighted MRI, were used to define the anatomical connectivity. Precision matrices were obtained with the region specific time series in five different frequency bands. We compared our method with the traditional GL and a functional adaptive version of the GL, in terms of log-likelihood and classification accuracies between the three groups. We conclude that introducing an anatomical prior improves the expressivity of the model and, in most cases, leads to a better classification between groups.

## Highlights

• We propose an anatomy-driven method for functional connectivity estimation in MEG.
• Structural prior contributes to a better representation of the functional connectivity.
• The proposed method is shown to be useful as a biomarker for classification of MCI.

## Introduction

The pre-dementia stage of Mild Cognitive Impairment (MCI) represents an intermediate state of cognitive decline that precedes the development of Alzheimer’s disease (AD) and other types of dementia (Petersen, 2011). The prevalence of MCI patients ranges from about 10% to 20% in people older than 65 years (Busse et al., 2006). From those that suffer from MCI there is a rate of progression to dementia of about 10% (Petersen, 2011). The pathophysiology of the disease lead to a progressive loss of synapsis efficacy (Selkoe, 2002) and loss of neurons as well as damage in the white matter, due to the phosphorylation of the Tau protein affecting axon transmission, and the accumulation of the beta amyloid protein, which impairs gabaergic transmission (Garcia-Marin et al., 2009). All these lead to the view of AD as a “disconnection syndrome” (Bajo et al., 2010, Delbeuck et al., 2003, Stam et al., 2009) in which a progressive damage of global functional and structural connections are potentially the cause of the insidious cognitive impairment. The functional consequences of this “disconnection syndrome” in MCI patients has been assessed with functional magnetic resonance imaging (fMRI) (Binnewijzend et al., 2012, Toga and Thompson, 2013, Wang et al., 2013a), electroencephalography (EEG) (Stam, 2003) and magnetoencephalography (MEG) (Bajo et al., 2012, Buldú et al., 2011, Zamrini et al., 2011).

The structural underpinnings associated with the functional disconnection may be studied in-vivo using diffusion-weighted magnetic resonance imaging (DW-MRI). Structural disconnection in MCI has been studied in terms of white matter integrity (Medina et al., 2006, O’Dwyer et al., 2011) and connectivity strength between pre-defined brain regions (Daianu et al., 2013, Shao et al., 2012). However, little is known about the relationship between the functional and structural components of brain network organization. A recent paper (Pineda-Pardo et al., 2014) showed that the functional impairment of resting-state MEG networks in MCI patients was related to the white matter (WM) integrity of specific tracts, thus pointing to a structural-functional relationship that could provide complementary information when studying this type of pathology.

The application of machine-learning classifiers to resting-state (“off-task”) functional connectivity (FC) is rapidly spreading, and is proving to be a valuable tool in the diagnosis of neurological and psychiatric pathologies (Atluri et al., 2013, Castellanos et al., 2013). The use of resting-state fMRI in the diagnosis of amnestic MCI was applied by Wee and colleagues (Wee et al., 2012b, Wee et al., 2012c). They achieved a 86% accuracy in patient classification, which was increased to 96% when including structural connectivity datasets.

The fact that structural and resting-state functional connectivity are strongly related has already been confirmed (Greicius et al., 2009, Van den Heuvel et al., 2009). However, the best way of combining these modalities to enrich our understanding of brain networks or to increase the diagnostic potential is still unclear. Computational models that simulate brain dynamics include the structural connectivity between nodes as coupling or constraining factors (Deco et al., 2013, Haimovici et al., 2013, Honey et al., 2007, Honey et al., 2009, Woolrich and Stephan, 2013). These models are capable of accurately reconstructing the large-scale networks derived from the slow fluctuations (< 0.1Hz) of fMRI data. There is also evidence that these models explain up to 40% of the functional connections that are observed with empirical MEG and fMRI datasets (Cabral et al., 2013, Honey et al., 2009). Diffusion-tractography informed priors have also previously been shown to improve inference of effective connectivity using Dynamic Causal Modelling of fMRI data (Stephan et al., 2009). Therefore we hypothesize that the structural connectivity may also increase the accuracy in the estimation of the FC for MEG data.

Traditionally empirical FC has been computed as linear correlations between time-series. However, correlation-based approaches only measure pairwise dynamics, and are unable to provide accurate topographies of the interactions between many brain regions (Smith et al., 2011). Also, standard correlation analysis produces fully connected functional networks, which are difficult to interpret. Furthermore, it is impossible to discriminate direct from indirect functional connections between two nodes, which can be driven by third nodes (Friston, 2011, Smith et al., 2011). The best way to overcome these limitations is to use biophysical models, such as Dynamic Causal Modelling, to infer effective connectivity (Friston et al., 2003). However, this becomes unfeasible for whole-brain connectivity analyses for both computational and statistical efficiency reasons.

Simpler models, such as the multivariate Gaussian distribution, where direct connectivity is modelled by means of the precision matrix, represent a useful alternative (Marrelec et al., 2006). The precision matrix is the inverse of the covariance matrix. Zero elements in this matrix represent an absence of direct connections, i.e., the partial correlation is zero. Regularization approaches based on the *l*_1_-norm (Vidaurre et al., 2013), such as the graphical lasso, allow one to find the zero elements in the precision matrix (Friedman et al., 2008, Smith et al., 2011), hence sparsifying the connection map, and, as a consequence, eliminating indirect functional connections. Sparse regression methods have been widely applied with fMRI time-series (Smith et al., 2011, Valdés-Sosa et al., 2005, Wee et al., 2013), however they typically do not consider DW-MRI structural connectivity information. One exception was the analysis by Hinne and colleagues (Hinne et al., 2014), where a shared adjacency matrix was estimated from an average across subjects of the structural connectivity. This matrix imposes a hard constraint on the precision matrix, completely defining the sparsity pattern before estimating the actual precision matrix. Inclusion of this sparsity constraint was observed to produce a significant improvement in the estimation of the FC compared with non-structurally informed FC.

In this paper, we present a flexible approach for including DW-MRI structural connectivity information in FC estimation based on resting state MEG data. In our approach, the structural connectivity guides (without strictly constraining) the precision matrix structure. This means that, for example, in the case of a questionable estimation of the structural connectivity, the model still has room to depart from the structural sparsity pattern. For this purpose, we include one adaptive penalization factor per connection. Unlike the approach used by Hinne and colleagues (Hinne et al., 2014), this adaptive factor is individualised for each subject. Although a similar approach has been employed using fMRI time-series obtained from healthy subjects (Ng et al., 2012), to our knowledge structural connectivity priors have not been previously applied to neurophysiological data such as MEG. We expect this approach to yield interesting results because, compared to fMRI data, MEG data is a more direct representation of the neural activity with finer temporal resolution, thus allowing us to study functional networks on a much wider frequency spectrum. Indeed, it has recently been shown that MEG can be used to infer appropriate FC in the resting state. However, FC is not computed as correlations on the raw time-series as it usually is done in fMRI, but over band-limited power time-series, particularly in the alpha and beta bands (Brookes et al., 2012, Luckhoo et al., 2012).

The aim of this work is hence to accurately estimate FC between brain regions, and to quantify how much the structural connectivity contributes to the estimation. We hypothesize that the element-wise adaptive penalization based on structural connectivity increases the accuracy in the estimation of the sparse networks. We compare this approach to cases in which we compute the adaptive penalization relying only on functional data, and in cases where the penalization is not adaptive. We also check whether the contribution of the structural connectivity to the functional connectivity estimation improves the accuracy of the discrimination between amnestic MCI subjects and healthy controls, and between the single-domain and multiple-domain subtypes of MCI. We carry out this analysis in different frequency bands. The accuracies are tested in a 10-fold cross-validation approach using four different classifiers: linear discriminant analysis (LDA), k-nearest neighbours (kNN), support vector machines with polynomial kernels (SVM), and support vector machines with radial basis functions kernels (SVMrbf). Consistently with the literature on other modalities, our results indicate that an appropriate inclusion of structural connectivity improves the classification.

## Materials and methods

**Fig. 1:**
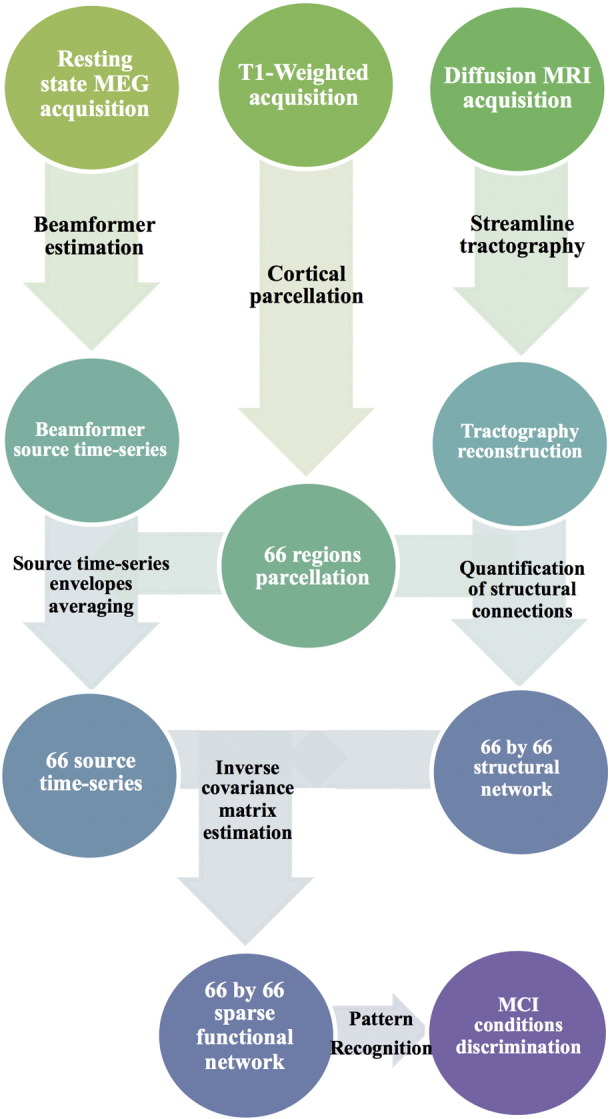
Flow-chart diagram describing the processing pipeline of the study, from the acquisition of MEG and MRI data to the statistical discrimination between healthy controls and MCI groups.

We next describe in detail our workflow, which comprises five parts: sample selection, MRI acquisition and analysis, MEG acquisition and analysis, estimation of the connectivity matrix and MCI condition discrimination. A summary in flowchart format can be found in Fig. 1.

### Sample selection

From an initial sample of 142 participants, we selected 81 due to: artifactual MEG dataset (N = 33); presence of vascular or tumour disease after structural MRI scans (N = 2); artifactual MRI datasets due to motion (N = 7); or due to unmatched ages between groups (N = 19). Twenty-nine subjects in the sample were healthy elderly controls (HC) that were recruited from the “*Seniors Center of the district of Chamartín, Madrid*”. The remaining fifty-two subjects were amnestic mild cognitive impairment (MCI) patients. Diagnosis of the MCI patients was reached through neuropsychological examination at the *Hospital Clínico de Madrid* and the “*UPDC del Ayuntamiento de Madrid*”. The diagnostic examination included: the Spanish version of the Mini Mental State Examination (MMSE) (Lobo et al., 1999), the Global Deterioration Scale (GDS) (Reisberg et al., 1982), the Functional assessment questionnaire (FAQ) (Pfeffer et al., 1982), the Geriatric Depression Scale (GDS) (Yesavage et al., 1982), the Hachinski Ischemic Score (Rosen et al., 1980), the questionnaire for Instrumental Activities of Daily Living (Lawton and Brody, 1969), and the Functional Assessment Staging (FAST) (Auer and Reisberg, 1997). MCI patients were classified at the stage 3 of the Global Deterioration Scale (GDS), and were diagnosed according to the criteria of Grundman et al. (2004) and Petersen (2004). All MCI patients showed memory complaints, abnormal memory functions, normal general cognitive functions (MMSE > 23), absence or minimal impairment in activities of daily living. They had no history of major psychiatric disorders or neurological diseases. None of the participants were medicated for their condition with cholinesterase inhibitors (e.g., donepezil) or other cognitive enhancing substances (e.g., memantine) before MRI and MEG scanning.

MCI patients were further divided in two groups, according to their clinical and neuropsychological profile. Single-domain MCI (sdMCI) showed isolated memory impairment, whereas multiple-domain MCI (mdMCI) showed a memory deficit accompanied by various degrees of impairment in cognitive domains such as executive functions, visuospatial skills, and/or language. A demographical description of the sample is included in Table 1. The groups showed no differences in age after t-test statistical comparison (p > 0.08). Also, no statistical differences were observed between groups in gender distributions after chi-squared statistical comparison (p > 0.16). However, revealed by a paired t-test (p < 0.01), there were differences in education scores and in mini-mental state examination (MMSE) evaluation.

**Table 1:** Demographic variables including gender, age, mini-mental state examination (MMSE) scores and education scores. Education level is quantified as: 1. Illiterate; 2. Elementary school studies; 3. Secondary school studies; 4. Technical or Mid-level studies; 5. Higher-education or University studies. Data are given as mean (standard deviation). ^1^p < 0.01 significant difference after paired t-test statistical evaluation in comparison between HC and sdMCI. ^2^p < 0.01 significant difference after paired t-test statistical evaluation in comparison between HC and mdMCI.

Group	Gender: Male/Female	Age	MMSE^1,2^	Education^2^
HC (N = 29)	8/21	71.52 (3.36)	29.21 (0.28)	3.44 (1.24)
sdMCI (N = 22)	10/12	73.00 (5.08)	27.80 (1.87)	2.73 (1.28)
mdMCI (N = 30)	7/23	73.06 (3.42)	26.77 (1.70)	2.53 (1.14)

The research described in this report was approved by the Ethics Committee of the *Hospital Clínico San Carlos*, Madrid. All of the participants signed a written informed consent before completing in any research activities.

### MRI acquisition and analysis

All images were collected using a General Electric 1.5 T magnetic resonance (MR) scanner, using a high-resolution antenna and a homogenization PURE filter. 3D T1-weighted anatomical brain MRI scans were acquired with a Fast Spoiled Gradient Echo (FSPGR) sequence with parameters: TR/TE/TI = 11.2/4.2/450 ms; flip angle 12°; 1 mm slice thickness, a 256 × 256 matrix and FOV 25 cm. Diffusion-weighted images (DWI) were acquired with a single-shot echo-planar imaging sequence with the following parameters: TE/TR 96.1/12000 ms; NEX 3 for increasing the signal to noise ratio (SNR); 2.4 mm slice thickness, 128 × 128 matrix and 30.7 cm FOV yielding an isotropic voxel of 2.4 mm; 1 image with no diffusion sensitization (i.e., T2-weighted b_0_ images) and 25 DWI (b = 900 s/mm^2^).

T1-weighted images were fed to Freesurfer (version 5.1.0) in order to segment each participant’s cortex into sixty-six anatomical cortical regions (Fischl et al., 2004) (see Supplementary Table 1 for region labels). These regions constituted the nodes for the anatomical and functional networks. Because of the low SNR in sub-cortical MEG source time-series, we used only cortical nodes. All segmentations were visually inspected, concluding that there was no need to discard any of the subjects of the sample due to incorrect grey matter segmentation. even in the presence of cortical atrophy (see Supplementary Fig. 1 for an example). Although omitting the connections to subcortical structures could potentially mislead the cortico-cortical connectivity, this atlas has still been proven to be useful in previous multimodal fMRI-DWI studies (Hagmann et al., 2008, Honey et al., 2009).

Diffusion-weighted images were pre-processed with FMRIB's Diffusion Toolbox (FDT-FMRIB Software Library v5.0). Pre-processing consisted of eddy-current correction, motion correction, and the removal of non-brain tissue using the Brain Extraction Tool (Smith, 2002). The diffusion tensor model was fit for the pre-processed diffusion images using least squares fitting with the Diffusion Toolkit Software (DTK v0.6.2). Tensor deflection tractography was applied to the diffusion tensor images to build the tractography (Lazar et al., 2003). Stopping criteria for the streamlines propagation were a maximum angle of 35° between consecutive steps and a lower fractional anisotropy, i.e. *FA* < 0.1 (Johansen-Berg et al., 2004). Only the tracts with a length larger than 15 mm were retained (see Supplementary Fig. 2 for an illustrative representation of the DWI temporal-signal to noise ratio (tSNR) and the performance of the tractography). The structural connection between a pair of nodes (*i*, *j*) was defined as the fiber density *FD*_*ij*_ of the connection, i.e. number of tracts touching the two regions divided by the total number of tracts, $\frac{n_{\mathrm{ij}}}{N}$. We normalized each of the connections by the average volume of the connected nodes, $FD_{\mathrm{ij}}=\frac{n_{\mathrm{ij}}}{N}\frac{2}{V_{i}+V_{j}}$, in order to control for differences in node size (Hagmann et al., 2008).

### MEG acquisition and analysis

MEG data were acquired with a 306-channel Vectorview system (Elekta-Neuromag) at the Center for Biomedical Technology (Madrid, Spain). The system comprises 102 magnetometers and 204 planar gradiometers on a sensor array, located inside a magnetically shielded room. Sampling frequency was 1 kHz, and an online anti-alias filter (0.1–330Hz) was applied. A head position indicator (HPI) system and a three-dimensional digitizer (FastrakPolhemus) were used to determine the position of the head with respect to the sensor array during the recordings. Four HPI coils were attached to the subject (one on each mastoid, two on the forehead), and their position with respect to the 3 fiducials (nasion, left and right pre auricular points) was determined. We recorded vertical eye movements, using two electrodes attached above and below the left eye in a bipolar montage. Resting-state acquisitions consisted of three-minutes recordings, where subjects were asked to stay calm and with their eyes closed. External noise was removed from the MEG data using the temporal extension of Signal-Space Separation (tSSS) (Taulu and Kajola, 2005) in MaxFilter (version 2.2, Elekta-Neuromag), using as parameters a window length of ten seconds and a correlation limit of 0.9. Participants’ head movements were corrected using the MaxMove extension of the software.

Electronic, muscle and ocular artifacts were automatically identified, and subsequently visually confirmed. The time series were segmented into trials of four seconds avoiding the segments containing any type of artifacts. Subjects with fewer than 15 clean trials were discarded. The mean number of clean trials was 25.9 ± 6.9 for the HC group, 27.8 ± 6.1 for the sdMCI group and 26.2 ± 5.5 for the mdMCI group.

Datasets were band-pass filtered in five frequency bands: alpha (8–13 Hz), low beta (13–20 Hz), high beta (20–30 Hz), full beta (13–30 Hz) and a broader band containing the theta, alpha and beta bands (4–30 Hz). The selection for these specific bands was performed in concordance with previous results in MEG, which show that it is the alpha band the most affected in amnestic MCI subjects (Garcés et al., 2013, Ishii et al., 2010) and in AD (Stam et al., 2009). Also, resting state fMRI large scale networks were shown to be reproducible in MEG data for alpha and beta bands (Brookes et al., 2011).

Padding segments of one second to the clean trials were included to avoid edge effects. The data covariance matrix was computed for each clean trial and then averaged for each frequency band. Instead of standard covariance matrix regularization, which is performed by adding uncorrelated noise (i.e. amplifying the diagonal of the covariance matrix) (Vrba and Robinson, 2000), we used a Bayesian principal component analysis (PCA) data dimensionality reduction, which has been demonstrated to work well in this particular domain (Woolrich et al., 2011).

The underlying currents of the time series observed in the sensor datasets were reconstructed using a linear constrained minimum variance beamformer (Van Veen et al., 1997). Lead-fields for all vertices in a 5-mm grid were obtained using a single-shell Boundary-Element-Model forward model (Mosher et al., 1999). Lead-fields for magnetometers and planar gradiometers were scaled following Mohseni et al. (2012) in order to allow the fusion of the sensor time-series. The source currents were estimated at each vertex of the grid covering the whole brain. Dipole orientations were estimated by searching for the maximum power projection of the dipole (Sekihara et al., 2001). Beamformer time-series were obtained by multiplying the beamformer coefficients by the band-pass filtered time series. These were subsequently normalized by the coefficients of variance as suggested in Hall et al. (2013) for the purpose of connectivity analyses. We followed previous work on resting state MEG FC (Brookes et al., 2012, Luckhoo et al., 2012) to obtain a single signal for each of the sixty-six cortical regions. For this matter, in order to avoid polarity swaps, we used the Hilbert transform to obtain the power envelopes of each time-series and then we averaged them within each region.

### Estimation of the connectivity matrix

We assume the data to have a Gaussian distribution, and we model the connectivity matrix as the estimated precision matrix, defined as the inverse of the covariance matrix. We denote the precision matrix as *Θ* and the sample covariance matrix as *S*. A zero in the precision matrix, say *Θ*_*ij*_ = 0, indicates that the corresponding partial correlation is zero, so that channels *i* and *j* are not directly connected (i.e., they are conditionally independent). The Gaussian assumption implies that dependencies between channels are always of second order, as higher order moments are always zero under this assumption.

In order to identify the connectivity pattern, we estimate a sparse precision matrix, i.e. with a number of elements exactly equal to zero. However, even if the covariance matrix is invertible (full rank), since data are always finite and noisy, the estimated precision matrix will have all elements different from zero. A popular way to get around these problems is to use *l*_1_-norm regularisation (Vidaurre et al., 2013), which provides both a numerically stable solution and a sparse estimation. Within the context of Gaussian inverse covariance matrix estimation, this is achieved through the graphical lasso (Friedman et al., 2008), which maximises the criterion

$$\log\det\left(\Theta \right)-\mathrm{tr}\left(\mathrm{S\Theta}\right)–\lambda \;\left||\Theta \right||{}_{1,}$$

where *λ* is the regularisation parameter and ‖ ⋅ ‖_1_ refers to the *l*_1_ -norm operator. To solve this problem, we use a coordinate-descent procedure that incorporates recent developments to accelerate computations (Witten et al., 2011).

It is well known that adaptive regularisation, which uses adaptive weights for regularising the different coefficients, improves the efficiency of the estimator and leads to more accurate sparsity patterns (Zou, 2006). An adaptive version of the graphical lasso can be readily obtained by maximising instead

$$\log\det\left(\Theta \right)-\mathrm{tr}\left(\mathrm{S\Theta}\right)–\lambda \;\left||W\cdot \Theta \right||{}_{1,}$$

where *W* is a matrix of weights with elements $W_{\mathrm{ij}}=1/\sqrt{S_{\mathrm{ij}}}$, and the operator ⋅ denotes element-wise multiplication. The same algorithm can be used to solve this problem. Thus, the adaptive setting is completely driven by the sample covariance matrix of the functional data.

In our approach, we assign values to *W*_*ij*_ based on structural connectivity information. In particular, we set $W_{\mathrm{ij}}=1/\sqrt{FD_{\mathrm{ij}}}$. By doing this, we inform the pattern of functional connectivity by a priori structural information in order to estimate more meaningful networks. We have implemented the three aforementioned flavours of the graphical lasso: non-adaptive graphical lasso (GL), functional data based adaptive graphical lasso (GLa), and structural data based adaptive graphical lasso (GLd). The computation of the Graphical Lasso was performed in R with the *glasso* Package,^1^ and we base on this to implement the adaptive varieties.

We used a 10-fold cross-validation to assess the methods in terms of log-likelihood and density of the networks. Within each fold we took the NIM11576 that minimizes the Bayesian Information Criteria (BIC), which amounts to choosing the model with the largest approximate posterior probability (Hastie et al., 2009). Model selection is performed within a routine in which we define an initial sequence of *λ* values. We estimate the precision matrices for each *λ* and compute the BIC statistic. We select *λ*_*min*_, which corresponds to the minimum BIC, and we define a new sequence of *λ* values within a relatively small vicinity of *λ*_*min*_. Following this procedure, we sharpen the search interval up to three times to obtain a final *λ*_*min*_. We limit the search to a maximum network density of 20%. This threshold is the number of non-zero links in the average of the structural networks across subjects, and is in general agreement with previous studies (Hinne et al., 2014). The final networks that were used to predict the conditions of MCI (see next Section 2.5) were obtained using the mean λ across the cross-validation folds.

Note that the BIC criterion needs an estimation of the effective sample size. When data are independent and identically distributed, this number equals the actual number of data points. In our case, each data point corresponds to a MEG measurement, so data is strongly autocorrelated. As a consequence, the effective sample size is lower than the number of data points. In this paper, we obtain the effective sample size by dividing the number of data points by one plus two times the sum of the autocorrelation values, for lags from 1 to a sufficiently high number – in practice, until the autocorrelation vanishes (Geyer, 1992). This is implemented in the function *ess* from the R *mcmcse* Package.^2^

### Pattern classification

We chose four different machine-learning classifiers to evaluate the accuracy of the predictions for the three network estimation methods: k-nearest neighbour (k-nn), linear discriminant analysis (LDA), support vector machine with polynomial (SVM), and radial basis functions (SVMrbf) kernels. Validation of the classification algorithms was performed with 10-fold cross-validation. For each run of the classification algorithms (e.g. one per graphical lasso approach, per frequency band, and per possible between-group combination), we performed a feature selection using non-parametrical Mann-Whitney statistical comparison between groups. The number of input features and the parameters of the classification algorithms described below were chosen by a nested 10-fold cross-validation procedure. The classification results of each fold were aggregated to the confusion matrix to obtain accuracies (rate of samples correctly classified), sensitivities (rate of samples in the second group correctly classified; see tables below), and specificities (rate of samples in the first group correctly classified).

LDA assumes that different groups generate observations based on different multivariate Gaussian distributions, so that, given two given groups, it is possible to define a boundary hyperplane where the probability for an observation to belong to any of the two groups is the same (Hastie et al., 2009). This boundary is then used to assign an observation to a group. We employed a regularized variant of LDA including a variable γ, in the interval [0,1], that attempts to shrink the group covariance matrices towards a diagonal matrix (Guo et al., 2007).

The k-nn classifier non-parametrically assigns an observation to the group to which the majority of the *k* closest training observations (nearest neighbours) belong (Hastie et al., 2009). The *k* closest neighbours were defined in terms of Euclidean distances, and *k* was chosen within the range [2,10].

SVM also defines a separating hyperplane in the feature space. The best hyperplane in this case will be the one with the largest margin between the two groups, where the margin is the distance between the closest samples to the hyperplane (Cortes and Vapnik, 1995). For the case of non-separable datasets, the margin is transformed to a soft margin, indicating that the hyperplane separates many but not all data points. Points in the feature space are typically mapped to some convenient space by means of the function *ϕ*(*x*) for which we only need to specify a kernel so that it holds *κ*(*x*, *x*′) = 〈*ϕ*(*x*), *ϕ*(*x* ′)〉, where (*x*, *x*′) are two instances in the feature space and 〈⋅〉 represents the dot product. For SVM, we employed polynomial kernels *κ*_*p*_(*x*, *x*′) = (1 + 〈*x*, *x*′〉)^*d*^ with *d* ranging from one to six. For SVMrbf, we used radial basis functions kernels *κ*_*rbf*_(*x*, *x*′) = exp(〈(*x* − *x*′), (*x* − *x*′〉)/2*σ*^2^), with *σ* taking values in 10^[− 5,− 4,…,4,5]^.

## Results

We first compare the ability of the different models to describe the data by reporting the cross-validated log-likelihood, which, assuming a Gaussian distribution, represents how well the precision matrix explains the (zero-mean) data set. Hence, the higher the log-likelihood, the more faithfully this model represents the data.

**Fig. 2:**
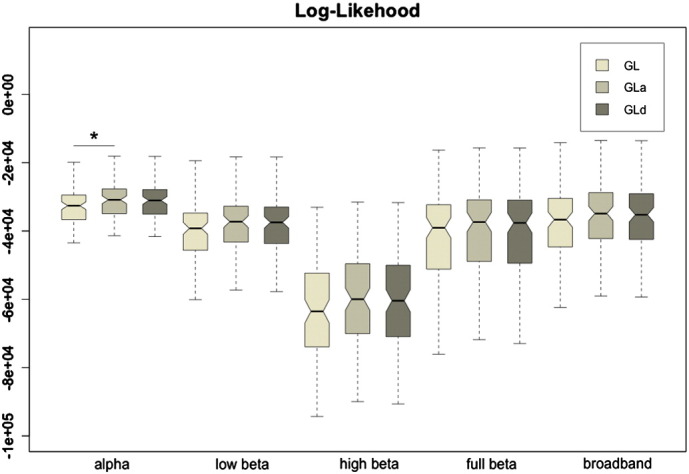
Boxplots of the log-likelihood values for the three graphical lasso approaches: GL, GLa and GLd. The asterisk indicates that the distributions differ with significance p < 0.05 after paired t-test statistical comparison.

Fig. 2 shows the average log-likelihoods for all three graphical lasso approaches and frequency bands. Although the adaptive versions, which introduce penalizations based on the inverse of the sample functional covariance matrix (GLa) or on the inverse of the structural connectivity weights (GLd), present very close likelihoods, they both are higher than the non-adaptive graphical lasso (GL). These differences are only significant in the alpha band (p < 0.05 after paired t-test statistical comparisons for GLa-GL), but were not significant after multiple comparisons correction using False Discovery Rate (FDR).

**Fig. 3:**
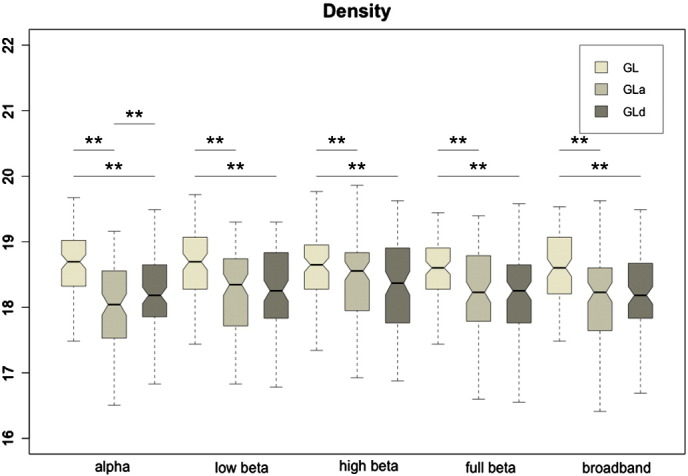
Boxplots of the density values for the three implemented graphical lasso approaches: GL, GLa and GLd. The double asterisk indicates that the distributions differ with significance p < 0.05 after paired t-test statistical comparison, and survived a False Discovery Rate multiple comparisons correction (qFDR < 0.05).

Fig. 3 shows boxplots with the densities (ratio of non-zero connections in the precision matrices) for all methods and frequency bands. Interestingly, the densities were higher for the GL than for the GLa or GLd. This, along with the above likelihood results, suggests that the adaptive approaches lead to more robust network topographies with fewer spurious connections. The differences were statistically significant (qFDR < 0.05) for GLa-GL and GLd-GL in all frequency bands, but, with the exception of alpha band, no statistical differences were observed between the GLa and GLd. In general, optimal densities for all methods and frequency bands were close to the maximum (20%).

For each type of functional connectivity dataset (GL, GLa and GLd), the aforementioned machine-learning classifiers were run to estimate clinical group predictions using 10-fold cross-validation.

In the classification between HC and sdMCI, the best performance was observed for LDA and SVMrbf classifiers, achieving up to 86% of accuracy. Table 2 shows the accuracies along with the specificities and sensitivities. The best classification results were obtained in alpha, high beta and broadband ranges of frequencies. The maximum achieved accuracy was of 86.27% (spec. 89.66% sens. 81.82%) obtained for GLd using broadband data. The classifier employed to obtain this accuracy was LDA and the chosen configuration parameters after the 10-fold cross-validation were 310 input features and *γ* = 0.5. The best accuracies for GL 82.35% (spec. 89.66% sens. 72.73%) and GLa 84.31% (spec. 89.66% sens. 77.27%) were obtained in the alpha band with SVMrbf and LDA classifiers respectively. Although these accuracies are close to the ones observed with GLd, it is clear that all four classifiers agree in that GLd with wide frequency band data offers the best predictor to discriminate between HC and sdMCI.

**Table 2:** 10-fold cross-validated accuracies for the classification between HC and sdMCI. The table shows the mean accuracies (specificities; sensitivities) in percentages for all five frequency bands, the three regularization methods (GL, GLa, GLd) and the four classifiers evaluated. Accuracies higher than 80% are highlighted.

HC vs sdMCI				
		GL	GLa	GLd
alpha	Knn	72.55 (79.31; 63.64)	72.55 (68.97; 77.27)	72.55 (72.41; 72.73)
	LDA	**80.39 (93.10; 63.64)**	**84.31 (89.66; 77.27)**	**82.35 (93.10; 68.18)**
	SVM	64.71 (75.86; 50.00)	76.47 (82.76; 68.18)	76.47 (86.21; 63.64)
	SVMrbf	**82.35 (89.66; 72.73)**	**80.39 (86.21; 72.73)**	**80.39 (93.10; 63.64)**
Low beta	Knn	72.55 (62.07; 86.36)	72.55 (65.52; 81.82)	68.63 (51.72; 90.91)
	LDA	70.59 (68.97; 72.73)	74.51 (75.86; 72.73)	72.55 (89.66; 50.00)
	SVM	66.67 (65.52; 68.18)	68.63 (75.86; 59.09)	66.67 (68.97; 63.64)
	SVMrbf	68.63 (72.41; 63.64)	72.55 (79.31; 63.64)	68.63 (72.41; 63.64)
High beta	Knn	70.59 (65.52; 77.27)	72.55 (79.31; 63.64)	74.51 (79.31; 68.18)
	LDA	**82.35 (86.21; 77.27)**	76.47 (79.31; 72.73)	76.47 (86.21; 63.64)
	SVM	66.67 (75.86; 54.55)	70.59 (75.86; 63.64)	**80.39 (86.21; 72.73)**
	SVMrbf	68.63 (93.10; 36.36)	76.47 (96.55; 50.00)	74.51 (86.21; 59.09)
Full beta	Knn	68.63 (62.07; 77.27)	70.59 (68.97; 72.73)	68.63 (62.07; 77.27)
	LDA	74.51 (82.76; 63.64)	68.63 (75.86; 59.09)	76.47 (82.76; 68.18)
	SVM	72.55 (79.31; 63.64)	64.71 (65.52; 63.64)	68.63 (75.86; 59.09)
	SVMrbf	76.47 (86.21; 63.64)	66.67 (96.55; 27.27)	72.55 (89.66; 50.00)
Broadband	Knn	74.51 (75.86; 72.73)	72.55 (79.31; 63.64)	**80.39 (72.41; 90.91)**
	LDA	78.43 (82.76; 72.73)	74.51 (82.76; 63.64)	**86.27 (89.66; 81.82)**
	SVM	74.51 (75.86; 72.73)	74.51 (75.86; 72.73)	74.51 (82.76; 63.64)
	SVMrbf	**80.39 (82.76; 77.27)**	72.55 (79.31; 63.64)	**82.35 (89.66; 72.73)**

**Fig. 4:**
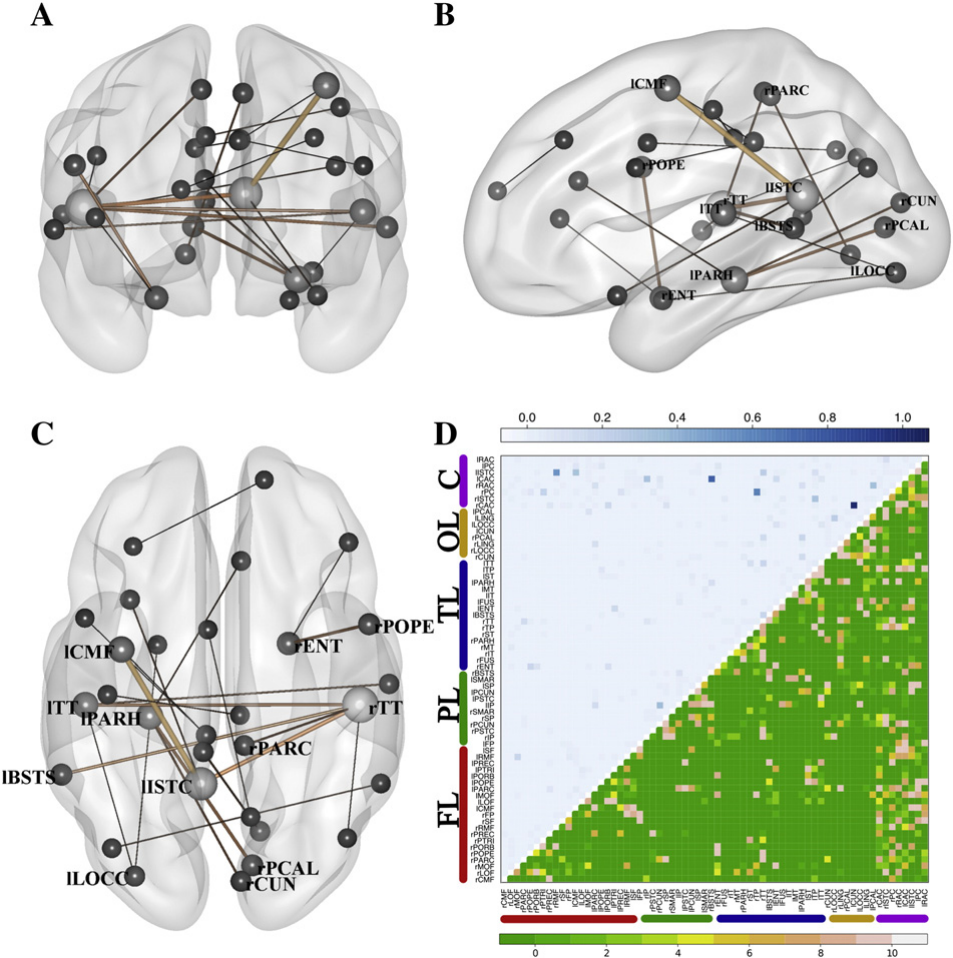
Selected features in the LDA classifier for HC-sdMCI for the broadband and GLd (accuracy 86.27%). A–C panels represent three views (sagittal, coronal and axial) of the selected links. The width and color (black to soft brown) of these links grows proportional to the median of the weights assigned to the links by the classifier across the ten folds of the cross-validation testing procedure. The size of the ball that represents the node is proportional to the number of links converging at this node. In panel D, the upper triangular matrix shows the median of the assigned weights and the lower triangular matrix represents the number of folds in which a specific link has been selected. The nodes were grouped according to brain lobes: FL – Frontal Lobe; PL – Parietal Lobe; TL – Temporal Lobe; OL – Occipital Lobe; C – Cingulate Cortex. See Supplementary Table 2 for a ranking of the links with the highest weights.

Fig. 4A−C represents, in a brain mesh, highly relevant connections that were selected by the previous LDA classifier in at least 9 of the 10-fold cross-validation testings. The width of the connections represents the median of the weights that the classifier assigned to this connection in each fold. For the sake of interpretation, only the top 1% of relevant connections are represented. Fig. 4D shows a matrix plot that represents in its lower part the number of folds that a specific link was selected and the median value assigned by the classifier across folds. The most important connections in this classification included temporal regions (right and left transverse temporal gyri; left entorhinal cortex; left parahippocampal gyrus), frontal regions (left caudal middle frontal gyrus), and cingulate regions (left isthmus of cingulate gyrus).

**Fig. 5:**
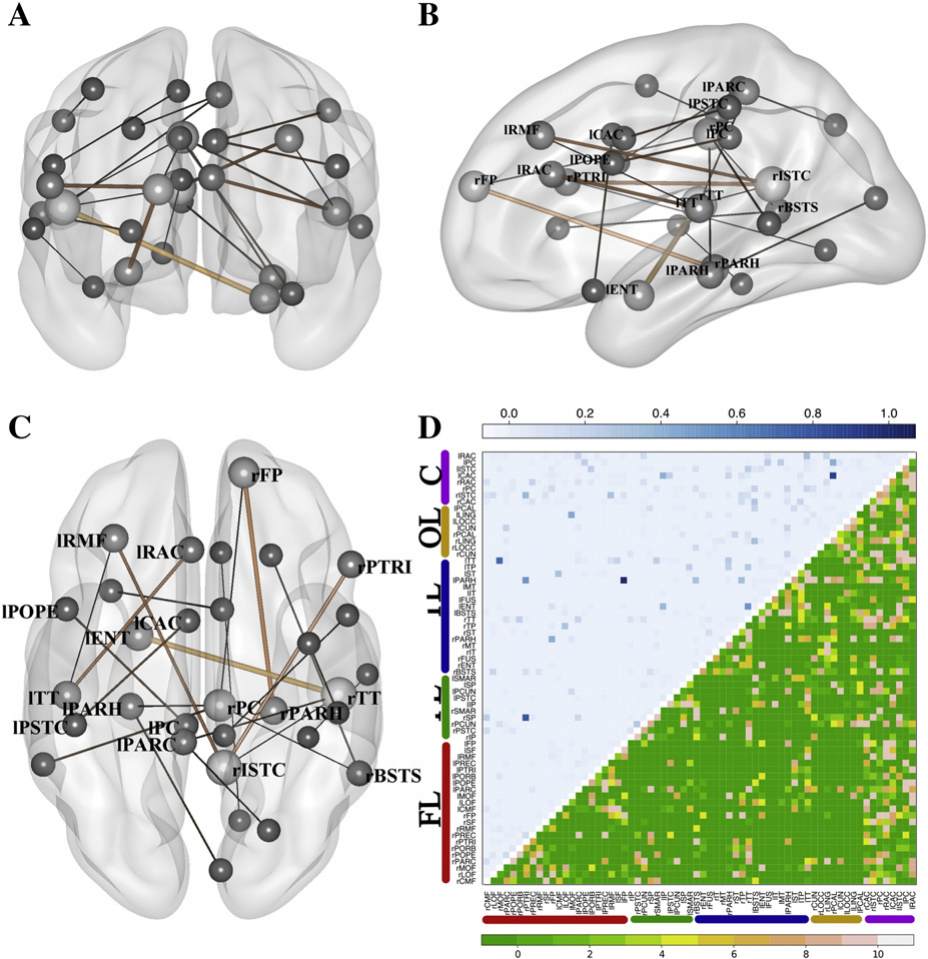
Selected features in the LDA classifier for HC-mdMCI in the full beta band for GLd (accuracy 81.36%). A–C panels are represent three views (sagittal, coronal and axial) of the selected links. The width and color (black to soft brown) of these links grows proportional to the median of the weights assigned to the links by the classifier across the 10-fold of the cross-validation testing procedure. The size of the ball that represents the node is proportional to the number of links converging at it. In panel D, the upper triangular matrix shows the median of the assigned weights and in the lower triangular matrix represents the number of folds in which a specific link has been selected. The nodes were grouped according to brain lobes: FL – Frontal Lobe; PL – Parietal Lobe; TL – Temporal Lobe; OL – Occipital Lobe; C – Cingulate Cortex. See Supplementary Table 2 for a ranking of the links with the highest weights.

Table 3 shows the accuracies in the classification between HC and mdMCI. The maximum achieved accuracy was of 81.36% (spec. 82.76% sens. 80.00%). This accuracy was achieved with GLd for the frequency band containing the full beta [13 − 30 Hz], and employing a LDA classifier with 385 input features and *γ* = 0.9. Figs. 5A–C depicts the most relevant selected features, following the same inclusion criteria described for Fig. 4.

**Table 3:** 10-fold cross-validated accuracies for the classification between HC and mdMCI. The table shows the mean accuracies (specificities; sensitivities) in percentages for all five frequency bands, three regularization methods (GL, GLa, GLd) and the four classifiers evaluated. Accuracies higher than 80% are highlighted.

HC vs mdMCI				
		GL	GLa	GLd
alpha	Knn	62.71 (62.07; 63.33)	64.41 (75.86; 53.33)	64.41 (75.86; 53.33)
	LDA	69.49 (75.86; 63.33)	76.27 (72.41; 80.00)	71.19 (62.07; 80.00)
	SVM	66.10 (48.28; 83.33)	66.10 (55.17; 76.67)	67.80 (58.62; 76.67)
	SVMrbf	59.32 (55.17; 63.33)	67.80 (72.41; 63.33)	64.41 (65.52; 63.33)
Low beta	Knn	69.49 (58.62; 80.00)	67.80 (75.86; 60.00)	62.71 (55.17; 70.00)
	LDA	71.19 (58.62; 83.33)	71.19 (72.41; 70.00)	76.27 (86.21; 66.67)
	SVM	66.10 (68.97; 63.33)	69.49 (68.97; 70.00)	66.10 (65.52; 66.67)
	SVMrbf	66.10 (79.31; 53.33)	69.49 (68.97; 70.00)	66.10 (72.41; 60.00)
High beta	Knn	59.32 (65.52; 53.33)	66.10 (65.52; 66.67)	64.41 (62.07; 66.67)
	LDA	62.71 (62.07; 63.33)	69.49 (68.97; 70.00)	71.19 (65.52; 76.67)
	SVM	55.93 (58.62; 53.33)	62.71 (58.62; 66.67)	66.10 (62.07; 70.00)
	SVMrbf	57.63 (27.59; 86.67)	59.32 (48.28; 70.00)	69.49 (65.52; 73.33)
Full beta	Knn	62.71 (79.31; 46.67)	62.71 (72.41; 53.33)	62.71 (72.41; 53.33)
	LDA	71.19 (72.41; 70.00)	76.27 (86.21; 66.67)	**81.36 (82.76; 80.00)**
	SVM	69.49 (75.86; 63.33)	64.41 (62.07; 66.67)	69.49 (65.52; 73.33)
	SVMrbf	62.71 (65.52; 60.00)	66.10 (62.07; 70.00)	67.80 (68.97; 66.67)
Broadband	Knn	66.10 (55.17; 76.67)	61.02 (86.21; 36.67)	62.71 (72.41; 53.33)
	LDA	67.80 (75.86; 60.00)	74.58 (82.76; 66.67)	71.19 (75.86; 66.67)
	SVM	61.02 (62.07; 60.00)	66.10 (75.86; 56.67)	61.02 (58.62; 63.33)
	SVMrbf	62.71 (65.52; 60.00)	66.10 (75.86; 56.67)	59.32 (62.07; 56.67)

The most relevant connections in the classification between HC and mdMCI were linked with temporal regions (right and left transverse temporal gyri, left entorhinal cortex, right and left parahippocampal gyri) and cingulate regions (right and left rostral anterior cingulate cortices, right isthmus of cingulate gyrus). These regions had also an important role in the classification between HC and sdMCI, where, besides, connections with frontal regions acquired more relevance. These include the right frontal pole, the left rostral middle frontal gyrus and the right pars triangularis.

**Fig. 6:**
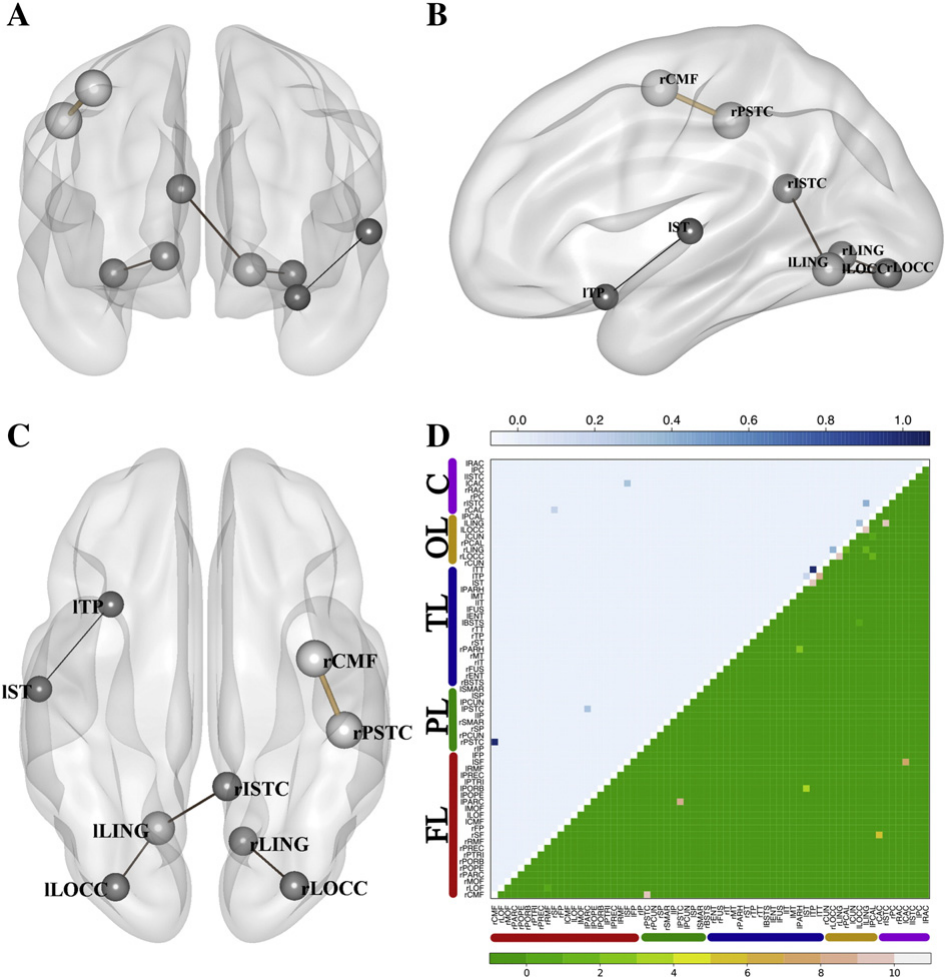
Selected features in the LDA classifier for sdMCI-mdMCI in broadband and with GL (accuracy 84.62%). Panels A-C represent three views (sagittal, coronal and axial) of the selected links. The width and color (black to soft brown) of these links grows proportional to the median of the weights assigned to the links by the classifier across the 10-fold of the cross-validation testing procedure. The size of the ball that represents the node is proportional to the number of links converging at it. In panel D, the upper triangular matrix shows the median of the assigned weights and the lower triangular matrix represents the number of folds in which a specific link has been selected. The nodes were grouped according to brain lobes: FL – Frontal Lobe; PL – Parietal Lobe; TL – Temporal Lobe; OL – Occipital Lobe; C – Cingulate Cortex. See Supplementary Table 2 for a ranking of the links with the highest weights.

In the classification between sdMCI and mdMCI, the LDA classifier showed the best outcomes (see Table 4). The maximum achieved accuracy was observed for GL in the broadband 84.62% (spec. 81.82% sens. 86.67%). This outcome was obtained with a LDA classifier with ten input features and *γ* = 0.5 (see Figs. 6 A-C for a graphical description of the most frequently selected features). In Fig. 6, many fewer links were selected for the classification between sdMCI and mdMCI. In this case, the maximum accuracy was achieved with only ten input features, which makes the number of depicted links (selected at least in nine of the ten folds) low. Note that the best accuracies for GLa and GLd were also obtained with LDA classifiers, and for the alpha and broadband datasets respectively.

**Table 4:** 10-fold cross-validated accuracies for the classification between sdMCI and mdMCI. The table shows the mean accuracies (specificities; sensitivities) in percentages for all five frequency bands, the three regularization methods (GL, GLa, GLd) and the four classifiers evaluated. Accuracies higher than 80% are highlighted.

sdMCI vs mdMCI				
		GL	GLa	GLd
alpha	Knn	59.62 (68.18; 53.33)	67.31 (54.55; 76.67)	65.38 (36.36; 86.67)
	LDA	71.15 (63.64; 76.67)	**82.69 (63.64; 96.67)**	78.85 (54.55; 96.67)
	SVM	57.69 (4.55; 96.67)	69.23 (59.09; 76.67)	63.46 (59.09; 66.67)
	SVMrbf	63.46 (18.18; 96.67)	76.92 (72.73; 80.00)	63.46 (27.27; 90.00)
Low beta	Knn	69.23 (50.00; 83.33)	67.31 (81.82; 56.67)	67.31 (77.27; 60.00)
	LDA	67.31 (45.45; 83.33)	69.23 (36.36; 93.33)	69.23 (54.55; 80.00)
	SVM	65.38 (50.00; 76.67)	59.62 (13.64; 93.33)	61.54 (22.73; 90.00)
	SVMrbf	65.38 (36.36; 86.67)	69.23 (59.09; 76.67)	61.54 (36.36; 80.00)
High beta	Knn	65.38 (45.45; 80.00)	63.46 (100.00; 36.67)	57.69 (90.91; 33.33)
	LDA	71.15 (68.18; 73.33)	69.23 (77.27; 63.33)	71.15 (72.73; 70.00)
	SVM	71.15 (36.36; 96.67)	67.31 (68.18; 66.67)	63.46 (50.00; 73.33)
	SVMrbf	67.31 (54.55; 76.67)	65.38 (27.27; 93.33)	61.54 (13.64; 96.67)
Full beta	Knn	63.46 (45.45; 76.67)	59.62 (40.91; 73.33)	69.23 (50.00; 83.33)
	LDA	67.31 (50.00; 80.00)	67.31 (36.36; 90.00)	69.23 (45.45; 86.67)
	SVM	63.46 (36.36; 83.33)	61.54 (18.18; 93.33)	65.38 (50.00; 76.67)
	SVMrbf	63.46 (36.36; 83.33)	59.62 (4.55; 100.00)	61.54 (9.09; 100.00)
Broadband	Knn	78.85 (72.73; 83.33)	71.15 (68.18; 73.33)	71.15 (59.09; 80.00)
	LDA	**84.62 (81.82; 86.67)**	78.85 (68.18; 86.67)	**80.77 (68.18; 90.00)**
	SVM	67.31 (54.55; 76.67)	63.46 (45.45; 76.67)	65.38 (45.45; 80.00)
	SVMrbf	78.85 (72.73; 83.33)	73.08 (50.00; 90.00)	76.92 (77.27; 76.67)

The most important connections in the classification between sdMCI and mdMCI included occipital regions (right and left lingual gyri; right and left lateral occipital gyri), cingulate regions (right isthmus of cingulate gyrus) and frontal regions (right caudal middle frontal gyrus, right postcentral gyrus). In this scenario, connections between temporal regions were not as diagnostic as they were before for HC-sdMCI and HC-mdMCI. This result is consistent with the cognitive state of these patients, as both groups sdMCI and mdMCI have a memory impairment. While in the classification between HC-sdMCI or between HC-mdMCI the GLd offers the best results, in the classification between sdMCI-mdMCI the best accuracies were obtained by GL (the non-adaptive case). In general, datasets in alpha and broadbands provided in general the best classification results. Consistent with previous reports, alpha is traditionally considered to be the main affected band in these amnestic MCI patients (Garcés et al., 2013, Ishii et al., 2010). However, we also provide evidence that the beta band also contributes to the classification, at least in those MCI patients with multiple impaired cognitive domains.

In summary, although the accuracies obtained using SVMrbf were high in general (in particular for the classification between HC and sdMCI), the best accuracies were obtained using LDA, suggesting that linearity is the best, most robust choice in this context. When it comes to the frequency contents, the alpha and broadband datasets got the best accuracies in the discrimination of sdMCI subjects, and the beta band gave the best performance in the classification between HC and mdMCI.

Besides the FC classification accuracies, we included the accuracies obtained from the power of the Hilbert-envelope time-series (see Supplementary Table 3) and from the FD matrices (see Supplementary Table 4). None of these results were better than 80% accuracy. The best results were obtained using the power of the envelopes in the broadband data as input features, reaching 78.43% (spec. 72.41%; sens. 86.36%) for the discrimination between HC and sdMCI. When using the FD matrices, the best results were 70.59% (spec. 72.41%; sens. 68.18%) for HC-sdMCI and 71.19% (68.97%; 73.33%) for HC-mdMCI.

## Discussion

In this paper, we have used three different varieties of the graphical lasso (Friedman et al., 2008), the non-adaptive graphical lasso, the functional adaptive graphical lasso, and the structural adaptive graphical lasso, to obtain sparse precision matrices explaining the direct functional connectivity between pairs of brain regions. To our knowledge, this is the first time that an adaptive sparse estimation has been applied to MEG data. The three approaches were evaluated in terms of log-likelihood, the network density, and the performance in the classification between three groups: healthy controls, amnestic MCI with a single (sdMCI) or multiple domains (mdMCI) affected.

In summary, we observed that, by including structural soft constraints, the classification accuracies were improved between MCI and HC groups in most cases. The best classification accuracies were obtained for the alpha and broadband datasets when discriminating sdMCI subjects. For HC and mdMCI, the full beta band achieved the best classification. Among the functional connections that were selected to discriminate between sdMCI and HC, we found several connections to temporal regions and to the posterior part of the cingulate cortex (Davatzikos et al., 2011, Sorg et al., 2007). The engagement of connections to frontal regions in the classification of mdMCI subjects agrees with the symptomatology of this condition (Schroeter et al., 2012).

### The contribution of structural connectivity

Single-modality biomarkers have been widely employed for the diagnosis of MCI or AD. As an advantage, they rely on simple imaging protocols, requiring less acquisition effort and costs. Nevertheless, we believe that the integration of information from different imaging biomarkers can considerably improve diagnosis and prognosis efficacy. There is evidence that structurally informed functional connectivity provides a more accurate representation of the real transfer of information that takes place in brain networks (Hinne et al., 2014, Ng et al., 2012). These approaches, however, have only been tested in healthy subjects, and have never been applied to the diagnosis of brain diseases. We demonstrate here that the inclusion of structural connectivity in the estimation model of functional connectivity improves the accuracy in the classification between MCI and HC: up to 3% of improvement for the sdMCI group (see Table 3) and up to 10% of improvement for the mdMCI group (see Table 4). In the classification between sdMCI and mdMCI, however, GL provides the best accuracy, perhaps because the structural connectivity differences are too heterogeneous, reflecting the variety of symptoms of the mdMCI group.

Our initial hypotheses were: first, that using the structural connectivity to guide the estimation of the network would improve the estimation of the functional connectivity; and second, that the classification accuracies between groups would benefit from this multimodal fusion. As mentioned above, the log-likelihoods quantify how well the model (in this case, the estimated precision matrix) describes the data under a certain distribution of probability (in this case, the multivariate Gaussian distribution). In our experiments, the log-likelihood increased when guided by the structural connectivity in comparison to the case of a homogeneous regularization. When comparing the log-likelihoods between the adaptive cases GLa and GLd, the results are mostly indistinguishable. GLa uses the empirical covariance matrix computed from the functional data in order to guide the estimation of the network and, hence, in terms of the ability of describing the functional data, it has a head start. The fact that the log-likelihood is roughly equal for GLd and GLa (and higher than GL) is indeed encouraging, considering that the adaptive penalty of GLa is defined using the same data modality (functional data) that we use for computing the likelihood. This result suggests a genuine link between functional and anatomical connectivity, consistent with findings elsewhere (Cabral et al., 2013, Greicius et al., 2009, Hinne et al., 2014, Honey et al., 2009).

### Disruption of functional networks in MCI

The classification of MCI using whole-brain connectivity is of growing clinical promise. Previous attempts distinguished between MCI that developed Alzheimer’s disease and HC with a 90% of accuracy (Shao et al., 2012) by using just structural connectivity. Here we obtained an accuracy of around 70% in the discrimination between groups when using only SC between cortical regions. This contrast of accuracies might be in part due to differences in the methodological pipeline. For example, we used deterministic tractography, and we did not included sub-cortical connections. Most likely, however, this is attributable to the advanced disease stage of the subjects, who would probably have had considerable cortical and sub-cortical atrophy that decreased the tract density, thus enhancing the discriminability power of the tractography.

In another study using graph-theory metrics from fMRI functional connectivity networks, Wee et al. classified amnestic MCI subjects versus HC with an accuracy of 86% (Wee et al., 2012b). This accuracy was increased to 96% when they concatenated functional and structural connectivity features (Wee et al., 2012c). These two studies had clear limitations: first, the reduced sample size (ten subjects in the sdMCI group) hinders the interpretation of the results; second, it is not clear whether the feature selection step was carried out on the entire data (including the testing data which is used to evaluate the model). In this case, this would result in an overestimation of the obtained accuracies. Also, leave-one-out cross-validation is known to be a high variance estimator of the classification accuracy (Hastie et al., 2009), and 10-fold cross-validation is a more reliable alternative (Kohavi, 1995).

The same group evaluated the classification performance in a similar sample using a group-constrained sparse estimation of fMRI connectivity through *l*_2_-regularization, achieving accuracies of 84% in the best case (Wee et al., 2014). In a third paper, they use sparse multivariate autoregressive modelling to compute an effective connectivity measure similar to Granger causality (Li et al., 2014). This time they achieved a mean accuracy of 91%, which turned out to be higher than those obtained using full-connected matrices (Pearson correlations). This result suggested that sparse functional networks provide a more effective representation of the whole brain connectivity. Note that fMRI is sensitive to other factors than brain activity, as the cerebral perfusion quantified as the cerebral blood volume/flow. Hypo-perfusion in regions such as the posterior cingulate has been related to cortical atrophy and cognitive decline in MCI patients (Chen et al., 2011, Lacalle-Aurioles et al., 2014). Thus, it is certainly possible that cerebral perfusion could also have contributed to the differences observed with the mentioned fMRI studies.

Note that not all of the patients included in our sample will necessarily develop Alzheimer’s dementia (non-converters), and, from those that will develop Alzheimer’s dementia (converters), the time to the conversion will likely vary. These two factors, as well as the possible development of other kinds of dementia, should be monitored on this sample, and the classifiers could be used to predict between an imminent development of dementia, a delayed development of dementia, and a return to the healthy condition.

The connections that most frequently distinguished both groups from the healthy control group include regions of the cingulate cortex that agree with those of previous findings in similar populations (Davatzikos et al., 2011). These regions were previously observed to have decreased functional connectivity in resting state networks derived from independent component analysis (Sorg et al., 2007). Connections with regions of frontal cortex were often selected, including caudal middle frontal gyri, rostral middle frontal gyri, frontal poles, and the pars triangularis (Grady et al., 2003). Interestingly, both HC-sdMCI and HC-mdMCI classifications assigned a similar importance to connections with regions of the temporal cortex, including transverse temporal gyri, enthorinal cortices and parahippocampal gyri. This seems reasonable, as the memory cognitive decline that both groups present might be related to a malfunctioning of regions in the temporal cortex. However, the importance in the classification of connections with frontal regions was much higher in the classification between HC-mdMCI. The fact that the mdMCI group presents a major executive impairment in comparison to the sdMCI group can motivate the role of this frontal connections as a clinical biomarker (Schroeter et al., 2012). It has also been reported that connections between frontal and temporal cortices are diminished in Alzheimer’s patients (Stam et al., 2009), i.e., at a more advanced disease stage. The medial frontal and temporal regions have been implicated in a “default-mode” network. This network comprises a set of regions with high connectivity during rest (Buckner et al., 2008), which become deactivated when performing any attention-demanding task. The level of activity in this network at rest has been observed to be lowered in individuals at risk of Alzheimer’s (Petrella, 2013, Vogelaere et al., 2012, Wang et al., 2013b).

In the classification between sdMCI-mdMCI, the maximum accuracy was obtained with ten input features. These connections included regions of the occipital cortex (i.e. lateral occipital and lingual gyri), cingulate cortex (isthmus of cingulate gyrus) and frontal cortex (caudal middle frontal and postcentral gyri). In other studies, the posterior part of the cingulate, including the isthmus cingulate, showed reduced metabolic activity in a set of patients that later developed Alzheimer’s dementia (Minoshima et al., 1997). Additionally to the posterior cingulate, the medial temporal lobe and the inferior parietal lobe showed decreased metabolic activity in AD patients (Gusnard and Raichle, 2001). This decreased metabolism has been found to correlate with pathological atrophy of the entorhinal and hippocampal cortices (Huang et al., 2002). According to Zhou et al. (2008), disruption of the anatomical connections from the posterior cingulate and the hippocampus to the whole brain in early AD patients were related to immediate recall memory scores. All these findings indicate that the posterior cingulate cortex is strongly involved in the course of AD, and the fact that connections with the isthmus cingulate are present in the classification between sdMCI-mdMCI might indicate that the connectivity with this region, as revealed by the proposed approaches, could be a useful connectivity-based biomarker to define the risk of converting to dementia.

For the sake of comparison, we have included classifiers using the power of the Hilbert envelopes time-series as input features. We observed that, in general, the accuracies were lower than those obtained using the graphical lasso approaches.

### Methodological issues and limitations

We should note a few limitations of this study. First, it is still unknown whether the MCI patients of this sample will convert to AD. This is important, as the classifier could lose predictive accuracy due to high heterogeneity within the groups. As an example, we evaluated this heterogeneity in terms of MMSE, as it seems that it could be a differentiating factor between groups (see Table 1). The misclassified subjects, however, were not significantly different from the rest of the group (i.e. the correctly classified subjects) according to a non-parametrical Mann-Whitney statistical comparison (p > 0.05) for any of the classifications.

Second, we have restricted ourselves to the alpha and beta bands. The selection of the frequency bands was made according to previous findings in MEG resting-state networks (Brookes et al., 2011) and to previous MEG literature in MCI (Garcés et al., 2013, Ishii et al., 2010). However, the impact of introducing structural priors for estimating functional connectivity in other classical frequency bands, such as delta (0.5–4Hz), theta (4–8Hz) and gamma (30–50Hz), was not evaluated and is still interesting. Future work should assess thoroughly the effect of structural priors in these frequency bands as well as the corresponding classification accuracies. The paper is also restricted to stationary measures of functional connectivity. Recent work using Hidden Markov Models (HMMs) has shown MEG resting state networks switching on very fast time-scales, on the order of 200 ms (Baker et al., 2014). Future work could assess the use of structural priors to constrain the cross-region interactions in the HMM observation models.

Third, the quantification of structural connectivity is prone to errors. Fiber crossing, bending or kissing are unresolved issues when using single DTI as a model of water diffusion. More complex models, such as q-ball (Tuch, 2004) or spherical deconvolution (Tournier et al., 2004) are potentially useful alternatives. However, the limited number of encoding directions in our data hinders this estimation. Future MRI protocols will include higher angular resolution diffusion schemes in order to achieve a better characterization of the structural connectivity.

Fourth, no standardized brain parcellation is fully agreed by the scientific community. There is evidence that different parcellation schemes give rise to different network topographies (Zalesky et al., 2010). The effects that this parcellation would have on the integration of structural and functional information is still an open question. Here, we employed a cortical anatomical parcellation of sixty-six regions which has been previously employed in multi-modal whole-brain network studies (Hagmann et al., 2008, Honey et al., 2009). In future analyses, we will test the impact of structural connectivity on the functional connectivity for different functional or anatomical parcellation schemes.

Fifth, we have not applied any leakage correction. Although there may be some benefits to using leakage correction due to removal of false positives (Maldjian et al., 2014), state-of-the-art methods are over-conservative in the sense that they remove all the zero-lag correlations (including the genuine ones), resulting in an (unknown) extent of true positives decrement. For this reason we considered that standard leakage correction could have unpredictable results in the context of precision matrix estimation and therefore chose not to apply it in these experiments. The inclusion of a structural prior, which is not contaminated by leakage, could have mitigated this effect as well. Note also that, despite this caveat (which is equally applicable to all the methods we compared), the results of the classification were still quite accurate. In the future, we will investigate the trade-off between true and false positives decrement by leakage correction, and, potentially, we will consider how to integrate leakage correction within the precision matrix estimation, perhaps by an inclusion of another adaptive penalty where closer links are more heavily penalised. This could be considered as a softer (less conservative) alternative to the usual orthogonalisations (Brookes et al., 2012, Maldjian et al., 2014).

Finally, the sample size of this study, although higher than previous investigations on HC-MCI discrimination (Shao et al., 2012, Wee et al., 2013, Wee et al., 2012c), is still moderate. Nevertheless, the presented results remain promising, and illustrate the potential of combining different modalities, for which we provide a simple but efficient method to carry through this integration.

## Conclusion

In this paper, we provide for the first time a multimodal integration of MEG-DWI for the estimation of sparse whole-brain networks. This integration has been compared in terms of classification accuracy between HC and MCI to two single-modality approaches: using the power of the Hilbert envelope time-series and single-modality SC. The conclusion is that GL-based techniques for estimating FC can yield higher accuracies than single modality approaches. The inclusion of SC in our FC estimation further improves the accuracies for HC-MCI discrimination but not for MCI subgroups classification. The reported accuracies are analogous to other results obtained from samples of amnestic MCI subjects using whole brain fMRI connectivity approaches (Wee et al., 2012a, Wee et al., 2012b, Wee et al., 2013). We conclude that whole brain MEG connectivity is a powerful biomarker of MCI and that the inclusion of SC increases the classification potential. Future studies with larger datasets and clinical follow-up will contribute to validate the effectiveness of the proposed integration.

## Footnotes

1 http://cran.r-project.org/web/packages/glasso.

2 http://cran.r-project.org/web/packages/mcmcse.
