# Supplementary material for: Guiding functional connectivity estimation by structural connectivity in MEG: an application to discrimination of conditions of mild cognitive impairment
Source: Neuroimage. 2014 Nov 1;101:765–77. doi: 10.1016/j.neuroimage.2014.08.002 (PMC4312351; doi:10.1016/j.neuroimage.2014.08.002)
Supplement: Supplementary file 1 — Supplementary material. [file mmc1.docx]

**Supplementary Material**

Sup. Figure 1. Freesurfer cortical segmentation in a subject with high atrophy. Panel A shows three views of the T1-weighted image. Panel B shows the cortical and subcortical segmentation masks. Panel C represents the cortical regions on a surface in the left hemisphere. Even though the ventricles show significant enlargement and the cortical thickness is reduced, the segmentation was satisfactory.

Sup. Figure 2. Diffusion weighted images for one subject. Panel A shows the temporal SNR (tSNR) across diffusion images. The values in the white matter range in the interval [3-11] and the values in the grey matter ranges in the interval [10-59]. Panel B shows the corresponding tractography. Panel C shows the tracts coming from the right precuneus.

| Bankssts | BSTS | Lingual | LING | Precentral | PREC |
| --- | --- | --- | --- | --- | --- |
| Caudalanteriorcingulate | CAC | Medialorbitofrontal | MOF | Precuneus | PCUN |
| Caudalmiddlefrontal | CMF | Middletemporal | MT | Rostralanteriorcingulate | RAC |
| Cuneus | CUN | Parahippocampal | PARH | Rostralmiddlefrontal | RMF |
| Entorhinal | ENT | Paracentral | PARC | Superiorfrontal | SF |
| Fusiform | FUS | Parsopercularis | POPE | Superiorparietal | SP |
| Inferiorparietal | IP | Parsorbitalis | PORB | Superiortemporal | ST |
| Inferiortemporal | IT | Parstriangularis | PTRI | Supramarginal | SMAR |
| Isthmuscingulate | ISTC | Pericalcarine | PCAL | Frontalpole | FP |
| Laterlaoccipital | LOCC | Postcentral | PSTC | Temporalpole | TP |
| Lateralorbitofrontal | LOF | Posteriorcingulate | PC | Transversetemporal | TT |

Sup. Table 1. Acronyms of the thirty-three cortical regions selected for analyses. These regions correspond to the grey matter parcellation proposed by Desikan et al. (2006).

| RANK | HC - sdMCI | HC - mdMCI | sdMCI - mdMCI |
| --- | --- | --- | --- |
| 1 | rCAC - lCUN | lPARH - lFP | lTP - lTT |
| 2 | rBSTS - lCAC | rPCAL - lCAC | rCMF - rPSTC |
| 3 | rPC - rTP | rPTRI - rSP | rISTC - lLING |
| 4 | rRMF - lISTC | rMOF – lTT | rLOCC - rLING |
| 5 | rPCUN - lIP | rTT - lENT | lLOCC - lLING |
| 6 | rRAC - lPCAL | rPTRI - lPARH | lCAC - lSF |
| 7 | lCMF - lISTC | lLING - lMOF | lPARC - lPSTC |
| 8 | rIP - lCAC | rPARH - rFP | rCAC - rSF |
| 9 | rISTC - lTP | rPCAL - lPC | lST - lTP |
| 10 | rLOCC - lLING | lCAC - lIP |  |
| 11 | rTT - lISTC | lENT - lIP |  |
| 12 | rPTRI - rPC | rISTC - rPTRI |  |
| 13 | rTT - lBSTS | rTP - lPARH |  |
| 14 | rPCAL - lPARH | lRAC - lTT |  |
| 15 | rMOF - lRMF | rLING - lBSTS |  |
| 16 | rENT - rPOPE | rBSTS - lPARH |  |
| 17 | rTT - lTT | rISTC - lRMF |  |
| 18 | rCAC - lPORB | lCMF - lST |  |
| 19 | lENT - lPTRI | rTP - lLOF |  |
| 20 | lCAC - lENT | rCAC - lBSTS |  |

Sup. Table 2. The twenty links that most contributed to the best classifications between HC-sdMCI, HC-mdMCI and sdMCI-mdMCI.

|  | | | | |
| --- | --- | --- | --- | --- |
|  |  | **HC vs sdMCI** | **HC vs mdMCI** | **sdMCI vs mdMCI** |
| **alpha** | **Knn** | 58.82 (62.07; 54.55) | 59.32 (44.83; 73.33) | 57.69 (68.18; 50.00) |
|  | **LDA** | 68.63 (72.41; 63.64) | 66.10 (65.52; 66.67) | 67.31 (45.45; 83.33) |
|  | **SVM** | 70.59 (65.52; 77.27) | 67.80 (62.07; 73.33) | 61.54 (59.09; 63.33) |
|  | **SVMrbf** | 70.59 (65.52; 77.27) | 67.80 (62.07; 73.33) | 61.54 (59.09; 63.33) |
| **Low beta** | **Knn** | 62.75 (48.28; 81.82) | 67.80 (65.52; 70.00) | 63.46 (77.27; 53.33) |
|  | **LDA** | 74.51 (72.41; 77.27) | 66.10 (58.62; 73.33) | 65.38 (59.09; 70.00) |
|  | **SVM** | 74.51 (72.41; 77.27) | 64.41 (65.52; 63.33) | 59.62 (59.09; 60.00) |
|  | **SVMrbf** | 74.51 (72.41; 77.27) | 64.41 (65.52; 63.33) | 59.62 (59.09; 60.00) |
| **High beta** | **Knn** | 56.86 (55.17; 59.09) | 61.02 (51.72; 70.00) | 61.54 (68.18; 56.67) |
|  | **LDA** | 72.55 (68.97; 77.27) | 64.41 (51.72; 76.67) | 67.31 (45.45; 83.33) |
|  | **SVM** | 68.63 (68.97; 68.18) | 59.32 (48.28; 70.00) | 67.31 (68.18; 66.67) |
|  | **SVMrbf** | 68.63 (68.97; 68.18) | 59.32 (48.28; 70.00) | 67.31 (68.18; 66.67) |
| **Full beta** | **Knn** | 62.75 (44.83; 86.36) | 61.02 (75.86; 46.67) | 63.46 (68.18; 60.00) |
|  | **LDA** | 72.55 (75.86; 68.18) | 64.41 (44.83; 83.33) | 69.23 (36.36; 93.33) |
|  | **SVM** | 66.67 (62.07; 72.73) | 62.71 (58.62; 66.67) | 61.54 (68.18; 56.67) |
|  | **SVMrbf** | 66.67 (62.07; 72.73) | 62.71 (58.62; 66.67) | 61.54 (68.18; 56.67) |
| **Broadband** | **Knn** | 68.63 (65.52; 72.73) | 66.10 (72.41; 60.00) | 63.46 (59.09; 66.67) |
|  | **LDA** | 76.47 (75.86; 77.27) | 69.49 (68.97; 70.00) | 67.31 (40.91; 86.67) |
|  | **SVM** | 78.43 (72.41; 86.36) | 69.49 (68.97; 70.00) | 59.62 (81.82; 43.33) |
|  | **SVMrbf** | 78.43 (72.41; 86.36) | 69.49 (68.97; 70.00) | 59.62 (81.82; 43.33) |

Sup. Table 3. 10-fold cross-validated accuracies for the classification between the three groups using as features the power of the sixty-six ROI Hilbert envelopes. The table shows the mean accuracies (specificities; sensitivities) in percentages for all five frequency bands and the four evaluated classifiers.

|  | **HC vs sdMCI** | **HC vs mdMCI** | **sdMCI vs mdMCI** |
| --- | --- | --- | --- |
| **Knn** | 70.59 (72.41; 68.18) | 69.49 (58.62; 80.00) | 65.38 (59.09; 70.00) |
| **LDA** | 68.63 (86.21; 45.45) | 67.80 (62.07; 73.33) | 71.15 (63.64; 76.67) |
| **SVM** | 68.63 (79.31; 54.55) | 71.19 (68.97; 73.33) | 61.54 (50.00; 70.00) |
| **SVMrbf** | 68.63 (79.31; 54.55) | 71.19 (68.97; 73.33) | 61.54 (50.00; 70.00) |

Sup. Table 4. 10-fold cross-validated accuracies for the classification between the three groups using as features the fiber density matrices. The table shows the mean accuracies (specificities; sensitivities) in percentages for the four evaluated classifiers.
